# Dietary intakes and food sources of fat and fatty acids in Guatemalan schoolchildren: A cross-sectional study

**DOI:** 10.1186/1475-2891-9-20

**Published:** 2010-04-23

**Authors:** Odilia I Bermudez, Claire Toher, Gabriela Montenegro-Bethancourt, Marieke Vossenaar, Paul Mathias, Colleen Doak, Noel W Solomons

**Affiliations:** 1 Tufts University School of Medicine, 136 Harrison Avenue, Boston, MA, USA; 2 Dublin Institute of Technology, Kevin Street, Dublin 8, Ireland; 3 Center for Studies of Sensory Impairment, Aging and Metabolism (CeSSIAM), 17 Avenida #16-89, Zona 11, Guatemala City, 01011, Guatemala; 4 Vrije Universiteit, Amsterdam, PO Box 7057, 1007 MB Amsterdam, the Netherlands

## Abstract

**Background:**

Consumption of healthy diets that contribute with adequate amounts of fat and fatty acids is needed for children. Among Guatemalan children, there is little information about fat intakes. Therefore, the present study sought to assess intakes of dietary fats and examine food sources of those fats in Guatemalan children.

**Methods:**

The study subjects consisted of a convenience sample of 449 third- and fourth-grade schoolchildren (8-10 y), attending public or private schools in Quetzaltenango City, Guatemala. Dietary data was obtained by means of a single pictorial 24-h record.

**Results:**

The percentages of total energy (%E) from total fat, saturated fat (SFA) and monounsaturated fat (MUFA) reached 29%E for total fat and 10%E for each SFA and MUFA, without gender differences. %E from fats in high vs. low-socio economic status (SES) children were significantly higher for boys, but not for girls, for total fat (p = 0.002) and SFA (p < 0.001). Large proportions of the children had low levels of intakes of some fatty acids (FA), particularly for n-3 FA, with >97% of all groups consuming less than 1%E from this fats. Fried eggs, sweet rolls, whole milk and cheese were main sources of total fat and, SFA. Whole milk and sweet bread were important sources of n-3 FA for high- and low-SES boys and girls, respectively. Fried plantain was the main source of n-3 FA for girls in the high-SES group. Fried fish, seafood soup, and shrimp, consumed only by boys in low amounts, were sources of eicosapentaenoic (EPA) and docosahexaenoic (DHA) acids, which may explain the low intakes of these nutrients.

**Conclusions:**

α-linolenic acid, EPA and DHA were the most limiting fatty acids in diets of Guatemalan schoolchildren, which could be partially explained by the low consumption of sources of these nutrients, particularly fish and seafood (for EPA and DHA). This population will benefit from a higher consumption of culturally acceptable foods that are rich in these limiting nutrients.

## Background

As many developing countries in the world are transitioning from high-carbohydrates diets to diets higher in fat, increases in prevalence rates of chronic conditions such as obesity diabetes and cardiovascular diseases are occurring. Dietary consumption of diets high in fats and in some fatty acids during childhood can have long-term health consequences. It is important for children to consume adequate amounts and types of fats, however, as they are essential components of healthy diets, insofar as linoleic (LA, 18:2n-6) and α-linolenic (ALA, 18:3n-3) acids are indispensable for functions in human anatomy and metabolism [1,2]. Thereafter, fat reserves in adipose tissues play a role in storage of lipid-soluble nutrients and thermal insulation of the body, whereas its oxidation yields metabolic energy along with carbohydrate and protein.

Data on fat intakes among children from industrialized countries reveal intakes higher than the recommended levels, which are targeted to contribute between 20 to 35% of total energy with no more than 10% of energy from saturated fats [3]. German school age children, 6-11 y, obtained 41% of their total energy from fats, and 20% of energy from saturated fats[4]. Studies with children from Italy [5], France [6] and the United States [7,8] also documented higher than recommended total and saturated fat intakes. Intakes of dietary fats among people from traditional societies exhibit different patterns, according to the demands of their environments. For example, Kuhnlein et al (2008) reported that traditional diets of Canadian adults from three indigenous groups (Inuits, Dene/Me'tis and Yukon First Nations), when compared to their recommendations for adequate intakes, are sufficient in n-3 PUFA but low in n-6 ALA [9]. In other settings, populations are in the midst of a nutritional transition [10], which is characterized by changes in the eating patterns. In Northern Mexico, children between 8-12 years of age of low socioeconomic status (SES) consumed diets high in total and saturated fats, and in cholesterol [11].

Although it is presumed that patterns of fat consumption are changing among Guatemalan children, there is little information to support this conjecture or to assess the direction of associations between fat intake and risk for chronic diseases risk. However, over the years, and with Guatemalan adults, results from dietary surveys have variously documented relatively low fat contributions to total energy on the order of 15% [12], 20% [13], or 27% [13,14]. There is also evidence that during the past 20 years, the Guatemalan population increased its consumption of dietary fats [15].

Evidence for the progression of nutrition transition has recently come from a combined anthropometric and dietary survey of 3rd and 4th grade schoolchildren in a provincial capital, Quetzaltenango City, Guatemala [16-18]. We present here the findings related to the selection and consumption of different food and beverages as major and minor sources of the fat intake of this population sample. We aimed at determining dietary intakes of fatty acids (FA) and the adequacy of those intakes, as compared to the World Health Organization (WHO) Dietary Goals [19]. We also sought to identify the main food sources of total fat and FA among our study sample.

## Methods

### Study population

Our study used data previously obtained as part of a cross-sectional study designed to assess nutritional status and dietary intakes of fruit and vegetable in a sample of 583 children, from 3rd and 4th grades in five public and seven private schools in the city of Quetzaltenango, in the western highlands of Guatemala. Detailed information about the original study had been documented elsewhere [16-18,20,21].

For the study reported here, we identified a sample of 449 children (48% boys, between 8-10 years of age) with complete dietary information. Using type of school, public or private, we classified children attending public schools as of low socio-economic status (LSES, n = 219) and those attending private schools as of high socioeconomic status (HSES, n = 230), similarly to criteria applied in previous publications based in this study [16-18,20,21].

The original survey was approved by the Human Subjects Committee of the Center for Studies of Sensory Impairment, Aging and Metabolism (CeSSIAM) and authorized by local education authorities. Informed assent from the children and informed consent from their parents were obtained. For this study, we also obtained approval from the Tufts Medical Center/Tufts University Investigation Review Board.

### Dietary data processing and analysis

Dietary data, collected during a 6 wk period between May and June 2005, was obtained with a single pictorial 24-hr recall complemented by a follow-on interview with a trained nutritionist, following a methodology developed and tested with schoolchildren attending public and private schools in Guatemala City [16-18,20,21]. Such methodology included the use of a 5-page booklet designed as the data collection instrument. Once informed consent from the parents was obtained, assenting children were asked to take a booklet home and to draw all foods and beverages consumed prospectively for a period of 24 h., both at home and at school. The instructions, explained by a research nutritionist, and contained in the booklet asked for details of all consumed items, including snacks, candies, brands and other similar characteristics. Once the recording time was completed, the research nutritionist interviewed the children. Completeness of the data was reviewed and portion sizes were estimated. In regards to foods eaten at schools, it is important to note here that no school lunch was provided by the participating schools and children brought their snacks from home or bought them at the school cafeterias. Schools in the target area end their school day at 1:30 pm and students return home for lunch.

Dietary data was processed with an initial review and codification of all food and beverage items. A total of 247 distinct foods and beverages were reported by the children. For the nutrient analysis of this data, we constructed a nutrient database, with complete data on fatty acids, based on the USDA food composition database, version 14[22], as no other nutrient database with information about fatty acid composition of foods was available in Guatemala. Our database included dietary lipids, cholesterol and principal fat classes, including saturated (SFA), monounsaturated (MUFA), polyunsaturated (PUFA), and individual fatty acids. Recipes for mixed dishes were created using information from the Latin American food composition tables from the Institute of Nutrition for Central America and Panama (INCAP), [23,24]. And we used proxy items from the USDA database to create equivalencies in nutrient content for local foods (mainly green leaves) without representation in the INCAP food composition tables.

### Statistical Analysis

We analyzed the study data with the Statistical Package for the Social Sciences, SPSS for Windows, release 16.0 (SPSS Inc., Chicago, Illinois). Preliminary analysis was firstly carried out to check normality of the data. We tested general linear models (GLMs) to assess levels of intakes of fats and fatty acids in children stratified by gender and SES, with adjustments for dietary energy (when appropriate) and for school grade.

For the assessment of adequacy of the intake of total fats (TF) and fatty acids, we compared intakes from the study sample of school age children with the dietary goals recommended by WHO [19]. Some of these dietary goals include setting limits in intakes of dietary fats as percent of total energy intake (%E) as follows: 15-30%E for TF, <10%E from SFA, PUFA as 6-10%E, n-6 FA as 5-8%E, and n-3 FA as 1-2%E [19]. Furthermore, WHO recommends an "optimal balance between intake of n-6 and n-3 polyunsaturated fatty acids" based on the recommended ranges above [19]. Based on those recommendations, we defined risk of inadequately high intakes of TF, SFA and n-6 as >30%E, ≥10%E and >8%E, respectively. PUFA <6%E, total n-6 <5%E and n-3 FA <1%E, were considered also as inadequately low levels of daily intakes. The ratio of n-6:n-3 FA was also assessed and a ratio of 8 or more was considered as inadequate. Additionally, intakes of cholesterol equal or above 300 mg/day were considered as excessive and inadequate. Results of these analyses were stratified for both socioeconomic class and gender, and evaluated logistic regression models, testing for the significance of the results between SES of the study subjects. All statistical tests were two-sided, and a p-value < 0.05 was considered significant. To determine food sources of dietary fats, we ranked foods by their contribution to the different nutrients reported here. Main food sources presented here stratified for both gender and SES.

## Results

### Intakes of fats and fatty acids

Intakes of dietary fats for the total sample reached 66 g of total fat, equal amounts (23 g) of SFA and MUFA and 14 g of PUFA (Table 1). Thirteen grams of n-6 FA and LA and approximately 1 g of both n-3 FA and ALA were also supplied by the diets of the study sample. When the mean intakes of those fats were adjusted by dietary energy, no differences were observed by gender. Intakes of EPA (0.009 g) and DHA (0.032 g) were higher in boys than in girls, even after energy adjustment. The n-6/n-3 ratio was 10.5 for the overall sample.

**Table 1:** Mean daily intakes (g/d) of fats and fatty acids by Guatemalan schoolchildren, by gender and SES

	High-SES		Low-SES		
		
Fats	Mean^1^	SE^1^	Mean^1^	SE^1^	P value^2^
**Total (n)**	230	--	219	--	--
Total fat (g)	65.7	1.0	64.6	1.0	0.435
SFA (g)	23.9	0.5	22.0	0.6	0.014*
MUFA (g)	22.9	0.4	22.5	0.4	0.486
PUFA (g)	13.3	0.3	14.5	0.3	0.005*
Total n-3 (g)	1.3	0.0	1.2	0.0	0.036*
Total n-6 (g)	12.0	0.3	13.3	0.3	0.001**
LA, 18:2n-6 (g)	11.9	0.3	13.2	0.3	<0.001***
ALA, 18:3n-3 (g)	1.2	.027	1.1	.027	0.045*
AA, 20:4n-6 (g)	0.12	0.01	0.13	0.01	0.736
EPA, 20:5n-3 (g)	0.010	0.002	0.008	0.002	0.455
DHA, 22:6n-3 (g)	0.032	0.002	0.032	0.002	0.819
Ratio n-6:n-3	9.6	0.20	11.5	0.20	<0.001***

**Boys (n)**	111	--	106	--	
Total fat (g)	68.7	1.5	63.4	1.6	0.002**
SFA (g)	25.1	0.7	20.3	0.8	<0.001***
MUFA (g)	23.9	0.7	22.3	0.7	0.083
PUFA (g)	13.7	0.4	15.1	0.4	0.036*
Total n-3 (g)	1.3	0.0	1.2	0.0	0.317
Total n-6 (g)	12.4	0.4	13.8	0.4	0.019*
LA, 18:2n-6 (g)	12.3	0.4	13.7	0.4	0.019*
ALA, 18:3n-3 (g)	1.23	0.04	1.19	0.04	0.440
AA, 20:4n-6 (g)	0.139	0.008	0.131	0.008	0.499
EPA, 20:5n-3 (g)	0.012	0.003	0.008	0.003	0.367
DHA, 22:6n-3 (g)	0.038	0.004	0.033	0.004	0.352
Ratio n-6:n-3	9.7	0.27	11.5	0.28	<0.001***

**Girls (n)**	119	--	113	--	
Total fat (g)	63.0	1.3	65.8	1.4	0.147
SFA (g)	22.9	0.8	23.6	0.8	0.488
MUFA (g)	22.0	0.6	22.7	0.6	0.416
PUFA (g)	12.9	0.4	14.0	0.4	0.060
Total n-3 (g)	1.3	0.0	1.2	0.0	0.055
Total n-6 (g)	11.6	0.4	12.8	0.4	0.027*
LA, 18:2n-6 (g)	11.5	0.4	12.8	0.4	0.018*
ALA, 18:3n-3 (g)	1.22	0.04	1.11	0.04	0.046*
AA, 20:4n-6 (g)	0.109	0.007	0.121	0.007	0.235
EPA, 20:5n-3 (g)	0.007	0.002	0.007	0.002	0.959
DHA, 22:6n-3 (g)	0.027	0.003	0.030	0.003	0.407
Ratio n-6:n-3	9.4	0.29	11.6	0.30	<0.001***

^1^Mean and standard error of the mean (SE) from general linear models, adjusted for energy intake

^2^Comparing adjusted mean intakes in children from high vs. low-SES, within gender groups. *P < 0.05; **P < 0.01; ***P < 0.001.

Abbreviations: LA: linoleic acid; ALA: alpha linolenic acid; AA: arachidonic acid; EPA: eicosapentaenoic acid; and DHA: docosahexaenoic acid

Boys from the high-SES group, as compared to those in the low-SES group, had significantly higher intakes of TF (p = 0.002) and SFA (p < 0.001) - see Table 1. Conversely, low-SES boys reported higher consumptions of PUFA (p = 0.036) and n-6 FA (p = 0.019), both total and as LA, relative to boys from the high-SES category. The n-6:n-3 ratio was also higher (p < 0.001) among boys in the low-SES group as compared to their peers in the high-SES group.

Intakes of fats were at the same levels between girls from both SES groups (Table 1). PUFA intakes were higher for the low-SES than the intakes observed in the other group (high-SES), although those differences only reached borderline significance (p = 0.06). However, intakes of n-6 FA, LA and ALA were significantly higher in the group of girls of low-SES category. As observed among boys, girls from the low-SES group presented higher (p < 0.001) intakes of the ratio n-6:n-3 as compared to girls in the high-SES group.

The contribution of fats to the total energy intake in the overall sample represented 30% from TF, about 10% from each, SFA and MUFA, 6% from each, n-3 FA and LA, with no detected differences by gender (Table 2). Daily intakes of cholesterol were 340 mg for the total sample, 377 mg for boys and 306 mg for girls, with significant gender differences (p < 0.05).

**Table 2:** Mean daily intakes (% of total energy intake) of fat and fatty acids by Guatemalan schoolchildren, by gender and SES

Dietary Fats	High-SES		Low-SES		P value^1^
		
	Mean	SE	Mean	SE	
**Total (n)**	230	--	219	--	--
Energy, kJ	8,324	176	8,174	180	0.554
Total fat, %E	29.6	0.42	28.6	0.53	0.149
SFA, %E	10.8	0.22	9.6	0.28	<0.001***
MUFA, %E	10.3	0.18	9.9	0.22	0.216
PUFA, %E	5.9	0.13	6.5	0.15	0.006**
Total n-3, %E	0.6	0.01	0.5	0.01	0.029*
Total n-6, %E	5.4	0.12	5.9	0.14	0.002**
LA, 18:2n-6, %E	5.3	0.12	5.9	0.14	0.001**
ALA, 18:3n-3, %E)	0.5	0.01	0.5	0.01	0.040*
AA, 20:4n-6, %E	0.060	0.003	0.061	0.003	0.843
EPA, 20:5n-3, %E	0.005	0.001	0.004	0.001	0.495
DHA, 22:6n-3, %E	0.016	0.001	0.015	0.001	0.644
Cholesterol (mg)	340	13	333	13	0.682

**Boys (n)**	111	--	106	--	
Energy, kJ	8,478	272	8,347	285	0.735
Total fat, %E	30.4	0.62	27.7	0.78	0.008**
SFA, %E	11.1	0.33	8.7	0.36	<0.001***
MUFA, %E	10.5	0.26	9.7	0.34	0.059
PUFA, %E	6.1	0.18	6.7	0.23	0.029*
Total n-3, %E	0.6	0.02	0.5	0.02	0.271
Total n-6, %E	5.5	0.17	6.2	0.21	0.015*
LA, 18:2n-6, %E	5.4	0.17	6.1	0.21	.014*
ALA, 18:3n-3, %E)	0.5	0.02	0.5	0.02	.436
AA, 20:4n-6, %E	0.068	0.004	0.062	0.004	0.348
EPA, 20:5n-3, %E	0.006	0.002	0.004	0.001	0.404
DHA, 22:6n-3, %E	0.020	0.002	0.016	0.002	0.196
Cholesterol (mg)	377	20.3	354	20.4	0.432

**Girls**	119	--	113	--	
Energy, kJ	8,318	234	8,150	230	0.611
Total fat, %E	28.8	0.56	29.4	0.71	0.485
SFA, %E	10.5	0.28	10.5	0.41	0.994
MUFA, %E	10.0	0.25	10.1	0.29	0.093
PUFA, %E	5.8	0.18	6.3	0.21	0.841
Total n-3, %E	0.6	0.02	0.5	0.02	0.052
Total n-6, %E	5.2	0.17	5.8	0.19	0.046*
LA, 18:2n-6, %E	5.2	0.17	5.7	0.19	.034*
ALA, 18:3n-3, %E)	0.6	0.02	0.5	0.02	.040*
AA, 20:4n-6, %E	0.053	0.004	0.060	0.004	0.227
EPA, 20:5n-3, %E	0.004	0.001	0.004	0.001	0.968
DHA, 22:6n-3, %E	0.013	0.002	0.015	0.001	0.414
Cholesterol (mg)	306	14.5	313	16.9	0.767

^1^Comparing mean intakes in children from high vs. low-SES, for the total sample and within gender groups, estimated with T-tests. Bold numbers indicate statistical significance between SES groups (*P < 0.05; **P < 0.01; ***P < 0.001).

Proportionally, children from the high-SES, as compared to those in the low-SES, obtained more energy from SFA (10.8% vs. 9.6%, p = 001) and n-3 FA (0.6% vs. 0.5%, p = 0.029) as seen in Table 2. Conversely, more energy was supplied by PUFA from diets of low (6.5%) vs. high (5.9%) SES children (p = 0.006). No significant differences in intakes of cholesterol were observed between the SES groups of children.

The intake of TF, as proportion of total dietary energy (%E) represented 30% and 28% for boys from the high and low-SES groups, respectively, as can be observed in Table 2. These differences were statistically significant (p = 0.008). Similarly, energy from SFA was proportionally higher (p < 0.001) for high-SES boys (11%) than for low-SES boys (9%). This last group, compared to high-SES boys, reported higher energy contributions from PUFA (7% vs. 6%, p = 0.029) and n-6 FA (6.2% vs. 5.5%, p = 0.015). Girls had similar energy contributions from TF and from all the fatty acids, except n-6 FA, for which low-SES girls had higher energy contribution relative to the high-SES group (Table 2).

The energy contribution of LA for the study subjects (total sample and stratified by sex) was significantly higher among those in the low SES vs. the high SES (Table 2). Conversely, the energy contribution of ALA was significantly higher in the high SES in the total sample, and in each sex category. For the total sample, the mean energy contributions of long chain PUFAs were similar, for both SES groups: approximately 0.6, 0.01% and 0.02% for AA, EPA and DHA. Similar values were observed when we examined the energy contribution of these long chain PUFAs within sex groups.

The most inadequate intakes of dietary fats were for n-3 FA, where more than 97% of the total sample as well as boys and girls failed to reach the lower end (1%E) of the recommended intakes of this nutrient (Table 3). Among the total study sample, we observed that, compared to children in the low-SES group, a significantly higher (p < 0.01) proportion of high-SES children consumed SFA, and n-3 FA at higher than recommended level. We also detected that almost half of the schoolchildren (total sample and sex groups) were at risk for low intakes of PUFA (<6% of total energy), plus more than a third of the evaluated groups were at risk for low intakes of total n-6 (less than 5% of total energy).

**Table 3:** Proportion of schoolchildren with inadequate intakes of fats^1^, by gender and SES

Nutrient	High-SES	Low-SES	OR^4^	95% CI^4^	
			
	%	%		Lower	Upper
**Total (n = 449)**	**n = 230**	**n = 219**			
Total fat >30 %E	49.1	43.8	0.82	0.56	1.19
SFA ≥10 %E	53.5	40.2**	0.59	0.40	0.86
PUFA >10 %E	3.5	7.8	2.30	0.97	5.45
PUFA <6 %E	53.9	45.7	0.70	0.48	1.02
Total n-6 <5%E	45.0	35.6*	0.65	0.44	0.96
Total n-6 >8 %E	7.4	16.4**	2.48	1.34	4.56
Total n-3 <1 %E	97.8	97.2	0.81	0.24	2.71
Ratio n-6:n-3 >8^2^	72.6	90.9***	3.90	2.26	6.76
Cholesterol ≥300 mg^3^	55.2	50.2	0.83	0.57	1.21
**Boys (n = 217)**	**n = 111**	**n = 106**	**--**	--	--
Total fat >30 %E	57.7	40.6*	0.5	0.29	0.87
SFA ≥10 %E	57.7	34.0***	0.37	0.12	0.65
PUFA >10 %E	3.6	10.4	3.06	0.94	9.98
PUFA <6 %E	53.2	41.5	0.61	0.36	1.05
Total n-6 <5%E	43.2	33.0	0.63	0.36	1.10
Total n-6 >8 %E	9.9	18.9	2.12	0.96	4.67
Total n-3 <1 %E	97.3	97.1	1.01	0.2	5.22
Ratio n-6:n-3 >8^2^	75.7	93.4***	4.79	1.97	11.68
Cholesterol ≥300 mg^3^	63.1	55.7	0.74	0.42	1.3
**Girls (n = 232)**	**n = 119**	**n = 113**	--	--	--
Total fat >30 %E	41.2	46.9	1.29	0.76	2.18
SFA ≥10 %E	49.6	46	0.88	0.52	1.48
PUFA >10 %E	3.4	5.3	1.58	0.43	5.78
PUFA <6 %E	54.6	49.6	0.80	0.48	1.35
Total n-6 <5%E	46.6	38.1	0.68	0.40	1.15
Total n-6 >8 %E	5	14.2*	3.13	1.18	8.33
Total n-3 <1 %E	98.3	97.3	0.63	0.1	3.84
Ratio n-6:n-3 >8^2^	69.7	88.5***	3.44	1.7	6.95
Cholesterol ≥300 mg^3^	47.9	45.1	0.91	0.54	1.54

^1^Based on WHO's dietary goals [19] as proportion of total energy intake. Risk for excessive intakes of TF, SFA, PUFA and n-6 were set at >30%, ≥10%, >10% and >8%, respectively. Low intakes of PUFA, n-6 and n-3 were set at below 6%, 5% and 1 %E, respectively.

^2^Ratio n-6:n-3 >8 was defined as inadequate

^3^For cholesterol, intakes equal or above 300 mg/day were considered as risk for excessive intakes.

^4^Differences, between SES groups, in prevalence of the risk for inadequate intakes of the fat and fatty acids listed in the table, were tested, for the total sample and within each gender group, with logistic regression models, with adjustment for energy intake in the case of cholesterol. OR = odds ratio; CI = confidence interval.

*p < 0.05; **p < 0.01; ***p < 0.001

Almost 60% of the high-SES boys consumed TF and SFA at or above the recommended upper levels (>30 %E and ≥10 %E, respectively); this was significantly higher than the respective proportion of boys from the low-SES group that had inappropriately high intakes of TF (41%) and SFA (34%), as detailed in Table 3. Low-SES boys were more likely to obtain higher proportions of energy from PUFA that boys in the high-SES group (odds ratio - OR = 3.1), although the differences between those two groups only approached statistical significance (p = 0.06). Among the girls, the proportions at risk for inadequate intakes of the fats and fatty acids reported in Table 3 were similar for those in both, the high and the low-SES groups, except for n-6 FA, where low-SES girls were 3 times more likely to get more than 8% of their energy intake from n-6 FA than their high-SES counterparts (OR = 3.1). Significantly more boys and girls of the higher SES presented a risk for unacceptable n-6:n-3 ratios than their peers in the low-SES strata (Table 3).

### Food sources of fats and fatty acids

The main foods sources of TF in diets of the Guatemalan schoolchildren studied contributed with 44% of the total intake and between 46% and 52% for the four groups stratified by gender and SES (Table 4). Fried eggs contributed with the larger proportion of total fat for the total sample, as well as for boys in both SES groups. Whole milk was the main source (8%) of total fat for girls in the high-SES, whereas sweet bread (8%) was the highest source of TF among girls in the low-SES. The proportional contribution of whole milk was higher for both boys and girls from the high-SES groups as compared to their counterparts in the low-SES groups. Other important sources of TF for the schoolchildren across gender and SES categories included fresh cheese, whole milk with sugar, fried potatoes, beef steak and hot dogs.

**Table 4:** Food sources of total fat (% of total intakes) in diets of schoolchildren, by gender and SES

Foods^1^	Total Sample	Boys (n = 217)		Girls (n = 232)	
		
		High-SES	Low-SES	High-SES	Low-SES
n	449	111	106	119	113
Fried eggs (%)	7.4	7.3	9.8	6.0	6.9
Sweet bread (%)	6.0	3.2	9.6	4.2	7.6
Whole milk (%)	5.0	6.7	3.6	7.8	4.0
Fresh cheese (%)	4.9	3.5	6.4	3.9	6.3
Fried potatoes (%)	4.4	5.3	3.9	3.7	4.6
Whole milk with sugar (%)	4.0	5.0	4.0	5.0	4.0
Beef steak, grilled (%)	3.8	4.9	3.5	3.5	3.3
Hot dog (%)	3.5	--	3.5	5.1	3.0
Black beans, cooked (%)	2.6	3.3	--	--	--
Fried plantain (%)	2.6	--	--	4.2	--
Pizza (%)	--	4.7	--	--	--
Cheese, **c**heddar (%)	--	2.7	--	--	--
Corn tortilla (%)	--	--	4.2	--	--
Fried **c**hicken (%)	--	--	3.4	--	--
Fried hamburger, homemade (%)	--	--	--	2.8	--
Hot chocolate from tablets (%)	--	--	--	--	5.0
Tomato gravy, spicy (%)	--	--	--	--	3.2

Subtotals (%)	44.2	46.6	51.9	46.2	47.9

^1^Foods listed were the main 10 contributors of total fat within stratified groups.

About one-fifth of the total intake of SFA came from whole milk (with or without added sugar) for high-SES boys and girls (Table 5), whereas fresh cheese was the first contributor of this nutrient for the total sample and the low-SES groups, 13% for boys and 11% for girls. Other food sources of SFA across gender and SES categories included fried eggs, beef steak, fried potatoes, hot dogs, pizza and sweet bread. For low-SES girls, hot chocolate represented more than 8% of the SFA intake.

**Table 5:** Food sources of saturated fat (% of total intakes) in diets of schoolchildren by gender and SES

		Boys (n = 217)		Girls (n = 232)	
		
Foods^1^	Total Sample	High-SES	Low-SES	High-SES	Low-SES
n	449	111	106	119	113
Fresh cheese (%)	8.9	6.1	12.5	6.9	11.2
Whole milk (%)	8.0	10.9	5.5	13.0	6.9
Whole milk with sugar (%)	8.0	9.0	7.0	9.0	7.0
Fried eggs (%)	5.9	5.5	8.4	4.6	5.4
Beef steak (grilled) (%)	4.3	5.2	4.3	3.9	3.7
Fried potatoes (%)	3.7	4.2	3.5	3.0	3.8
Hot dog (%)	3.6	2.5	3.9	5.2	3.0
Pizza (%)	3.3	7.0	--	3.9	--
Sweet bread (%)	3.2	--	5.6	--	4.0
Hot chocolate (%)	3.0	--	--	--	8.3
Cheese, American (%)	--	4.7	--	4.0	--
Black beans, oil added (%)	--	3.5	3.9	--	--
Refried beans (%)	--	--	2.2	--	--
Fried hamburger, homemade (%)	--	--	--	3.1	--
Donuts (%)	--	--	--	--	3.5

Subtotals (%)	51.9	58.6	56.8	56.6	56.8

^1^Foods listed were the 10 main contributors of saturated fat intakes within stratified groups.

**Figure 1:**
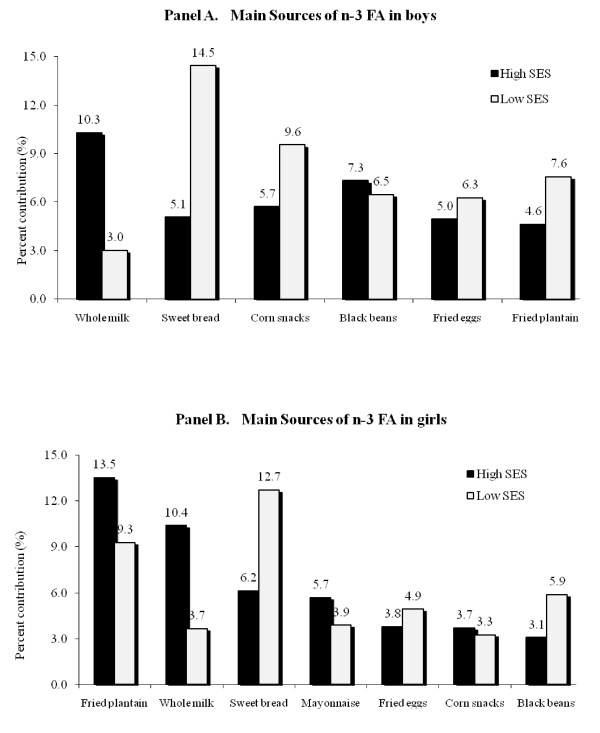
**Main sources of n-3 FA in boys (panel A) and in girls (panel B)**.

On the one hand, low-SES boys and girls depended mainly on sweet bread as their main source of n-3 FA (Figure 1, panels A and B), while boys from the high-SES obtained these fatty acids from whole milk, cooked black beans and sweet bread. On the other hand, high-SES girls had fried plantain, whole milk and mayonnaise as their main sources of n-3 FA.

**Figure 2:**
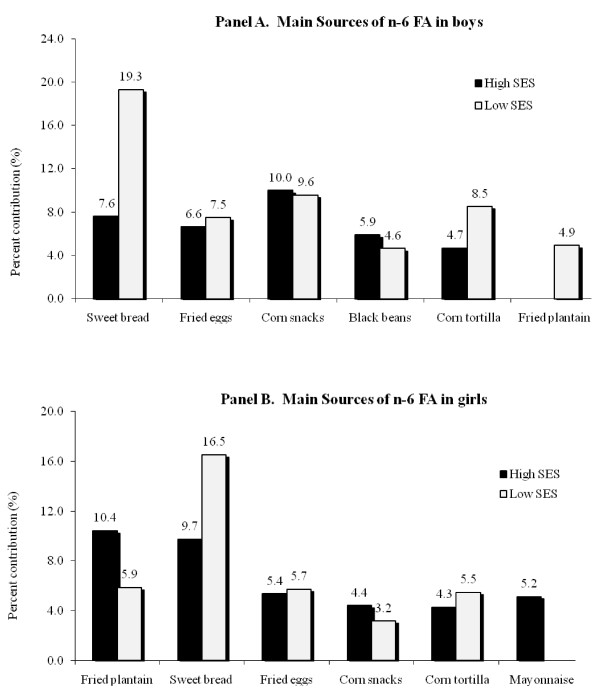
**Main sources of n-6 FA in boys (panel A) and in girls (panel B)**.

The n-6 FA were obtained mainly from corn-based snacks, sweet bread, fried eggs and corn tortilla in diets of the high-SES boys (Figure 2, panel A). For their peers in the low-SES group, about 20% of the total intake of n-6 FA came from sweet bread and about 10% from corn-based snacks. Low-SES girls (Figure 2, panel B) also obtained most of their n-6 FA from sweet bread, while those girls in the high-SES segment got these nutrients from fried plantain, sweet bread and fried eggs.

**Figure 3:**
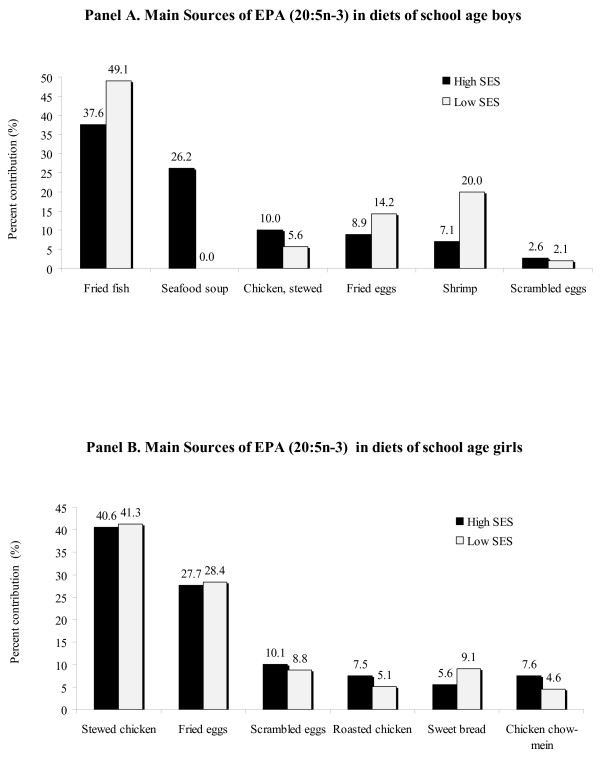
**Main sources of Eicosapentaenoic acid (EPA, 20:5n-3) in boys (panel A) and in girls (panel B)**.

**Figure 4:**
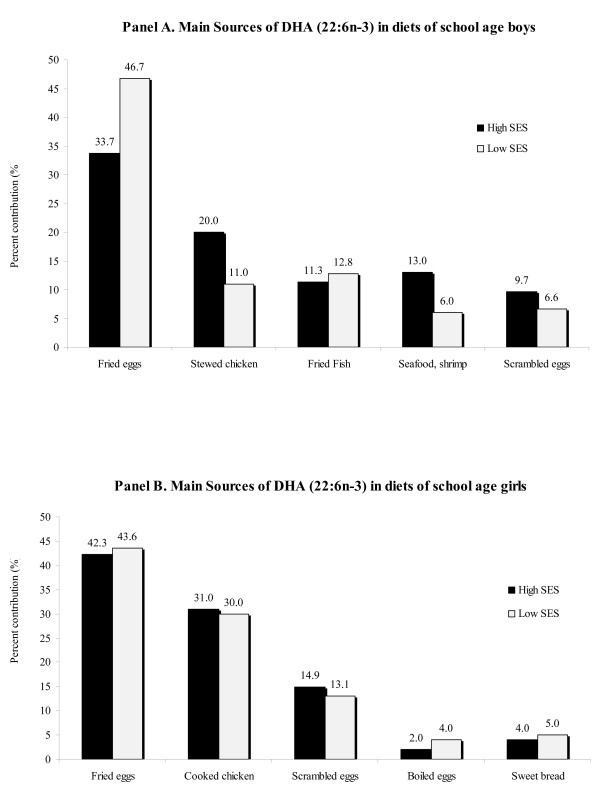
**Main sources of Docosahexaenoic acid (DHA, 22:6n-3) in boys (panel A) and girls (panel B)**.

Fried fish and seafood soup (only for high-SES boys), fried eggs and shrimp for boys from both SES groups were the main sources of EPA, as represented in Figure 3, panel A. For girls in both SES groups, however, stewed chicken, fried eggs and scrambled eggs were the principal contributors of EPA (Figure 3, panel B), noticing the absence of fish/seafood among the girls (these food did not appear in the recall data). As seen in Figure 4, foods identified as important contributors of DHA in boys (panel A) and girls (panel B) included fried eggs, chicken and scrambled eggs, with fried fish and shrimp contributing, but only for boys.

## Discussion

Based on information collected with a pictorial dietary recall instrument, complemented with additional interview with a research nutritionist, we were able to characterize daily intakes of dietary fats intakes in diets of school age children from the highlands of Guatemala. We also constructed a database with fatty acid values which was an important contribution to Guatemalan researchers exploring dietary behaviors around fats. In this study, we report for the first time intakes of essential fatty acids in diets of school age children from Guatemala.

The appropriateness and safety of limiting total energy from fat to an upper limit of 30% to young children is still debated [25-28]. However many well controlled studies have concluded that current recommendations for dietary fat are sufficient to ensure adequate growth, and nutritional status in childhood, while diets higher in fats may result in increased body fat [29-31]. We found that high proportions of children in our study, particularly those in the high-SES groups, were at risk for excessive intakes of TF and SFA, as well of cholesterol; along with large proportions of children with low intakes of n-3 FA. Although the mean TF and SFA reported here for school age children, stratified by gender and SES, were lower compared to the mean intakes of TF (81 g/day) and SFA (31 g/day) reported for American children [32].

Urbanization, modernization and global food trade have been responsible for an increase in the contribution of fat to diets in low-income countries, as part of nutrition transition [10]. As traditional populations modernize their dietary patterns, they tend to change in a somewhat stereotypical manner [10], with increases in animal items, which include largely SFA of animal origin, with a concomitant reduction in the unsaturated fats of plant, whereas separated fats and oils are consumed in increasing amounts. Children are an interesting group to focus on, as there is uncertainty in the interpretation of lipid patterns in this age group and the need for prophylactic response. Recently, the American Academy of Pediatric has validated the use of cholesterol-lowering medications for children as young as 8 y., the lower limit of the present population sample [33].

At the time of our survey, in 2005, neither the price pressure on maize markets from the dual directions of diversion to biofuel and increased transport-related costs from escalating petroleum prices had appeared, suggesting that food insecurity had not yet arrived. Groeneveld et.al [16], findings on overweight prevalence of 12.9% in low, and 32.1% in high-SES in these schoolchildren also attests to a lack of widespread caloric constraint at the time of the survey. We would also conclude that no barriers to modernization of food practices were in operation at the time.

The study has a number of strengths and a series of acknowledged caveats and limitations. The recent release by the USDA of a complete food composition database for all fatty acid species in foods and beverages permitted such complete differentiation heretofore not possible for survey analyses. We have used a recently released and complete food composition database for fats, which differentiates fatty acids more completely than any predecessor [22]. Insofar, however, as our subjects consumed typically Guatemala recipes with no representation in the food tables, we had to create equivalencies based on proxy items that bore similarities to the local dishes.

The survey approach used self-reporting by children as the data-collection strategy, with the limitations that we address here. The generic issues of precision and accuracy of 24-h recalls are widely understood[34]. Unfortunately, due to logistic constraints, we were not able to repeat the intake record, even in a subset of children, in order to make any correction for the true variance of distributions. Therefore, to respect these limitations, particularly the disadvantage of the single record data not being representative of the usual nutrient intake at the individual level, we limited our analysis at the group and subgroup (e.g. SES and sex) levels.

Given the problems in calibration single-day intake recording to other dietary intake methods, 24-h methods are challenging to validate. Favorable opinions on children's abilities to report their own findings have been published [35-40]. The innovative, pictorial diet record approach applied in this study represents an innovation to improve memory [34,39], and the nutritionist's probes in household measures serves to better gauge amounts.

## Conclusions

In this study we reported the dietary patterns of Guatemalan schoolchildren with respect to fat and fatty acids, which is the first report of fatty acid consumption in children from this country. Contrary to what reported intakes among children from North America and European countries [4-8], intakes of fat and fatty acids, particularly total fat and SFA, were much lower than the reported values in those studies, and, therefore, closer to the WHO recommendations for these nutrients [19]. However, as we documented here, the consumption of fish and other seafood was very low among boys, although they represented the important sources of EPA and DHA. These foods were non-existent among girls. The low (boys) or null (girls) intakes of fish/seafood partially explain the low intakes of EPA and DHA, which along with ALA, were the essential fatty acids found to be the most limiting in the diets Guatemalan schoolchildren. This population will benefit from a higher consumption of culturally acceptable foods that are rich in these limiting nutrients. The fact that boys reported consumption of fish and other seafood is an indicative that these foods are acceptable options for Guatemalan children.

## Competing interests

The authors declare that they have no competing interests.

## Authors' contributions

OIB, CD, PM and NWS developed the research protocol. GMB conducted the data collection. CT, GMB, and MV were responsible for data analyses. OIB and NWS drafted the paper. All authors critically reviewed the manuscript and approved the final version submitted for publication.
